# Update on Campbell's Countering Violent Extremism programme

**DOI:** 10.1002/cl2.1387

**Published:** 2024-02-29

**Authors:** Peter Neyroud, Ajmal Aziz, Brett Kubicek

**Affiliations:** ^1^ Institute of Criminology University of Cambridge Cambridge UK; ^2^ Department of Homeland Security Washington District of Columbia USA; ^3^ Canada Centre for Community Engagement and Prevention of Violence, Public Safety Canada Ottawa Ontario Canada

The Campbell Countering Violent Extremism (CVE) evidence synthesis programme is a global research initiative focused on using rigorous and relevant evidence to inform policy and practice. The programme was designed to produce and publish a series of high‐quality Campbell systematic reviews and evidence maps in priority areas agreed in consultation with the Five Research and Development (5RD) Countering Violent Extremism Network (CVEN). This multilateral partnership of government home affairs/interior departments from Australia, Canada, New Zealand, United Kingdom and United States aims to share scientific research and evidence‐based knowledge to ensure that participant nations are better prepared to divert individuals from radicalisation to violent extremism and terrorism, to prevent individuals from carrying out attacks, to mitigate the impact of violent extremist and terrorist events, and to develop community and individual resilience to these kinds of targeted, grievance‐fuelled violence. This multilateral partnership is able to bring in other bilateral (e.g., Sweden) and multilateral (e.g., Five Country Ministerial) entities to help collectively expand the adoption of evidence‐based approaches to countering violent extremism.

The CVEN was established in 2015 to provide a forum to enable broad‐ranging cooperative Research, Development, Test & Evaluation (RDT&E) among public safety and security entities with a goal of connecting efforts from within governments, academia, and the private sector to enable forward‐thinking CVE, and terrorism and threat prevention, while leveraging global expertise in a single forum. On side of other fields of violence and harm prevention, the field of CVE is still relatively new. Further, given the focus on low frequency, high‐consequence events, the field has had challenges in building up data and evidence to support the assessment of trends, risks, needs, vulnerabilities and protective factors, as well as for evaluating what approaches to prevention work for whom in what context. As such, a central aim for the CVEN is to support coordinated investment to address these gaps, and raise the bar on standards of evidence and practice.

The partnership between CVEN and the Campbell Collaboration Crime and Justice Coordinating group has run for 4 years, with four cycles of topic development and commissioning of reviews. This time commitment has enabled a learning process in how to bring systematic evidence reviews into a relatively nascent field of prevention, with innovations including to develop protocols for reviewing qualitative studies, a key step given the importance of context in the design and implementation of CVE programmes. The long‐term commitment is also helping ensure that the growing number of empirical studies relevant for CVE have a stronger foundation on which to build. In the sections below, we present main findings of the key Campbell reviews which have been completed and published so far.

## 1 POLICE PROGRAMMES TO GENERATE COMMUNITY CONNECTEDNESS

The first to be published was Mazerolle et al.'s (2020) review of police programmes that seek to generate community connectedness, a review that set out most directly to answer the question about the effectiveness of community policing in this context. These programmes are assumed to help enhance protective factors and reduce risk factors that lead individuals to radicalise to violent extremism. The review found that there is no robust body of evaluation evidence to verify this claim. Although the systematic search captured 2273 potential studies, only one study met the review inclusion criteria (a controlled design study with a robust design and low risk of bias).

That one study (Williams et al., 2016) was conducted in the USA and assessed the effectiveness of the WORDE model of prevention. This model, described by Mirahmadi (2016), proposed four elements:

- Engaging a wide range of stakeholders with a view to supporting a cohesive community neighbourhood.
- Educating the stakeholders to be an ‘informed and aware citizenry dedicated to public safety’ (p.137).
- Create mechanisms to connect vulnerable individuals to support resources.
- A network of professional and community resources capable of responding to the referrals.

Williams et al. (2016) found that the programme had delivered on its initial implementation ambitions and had fostered positive social integration. Mazerolle et al. (2020) concluded that the ‘evidence from the study showed mixed small‐to‐medium effects on self‐reported deradicalization measures in favour of the treatment group’ (p.1). One survey item favoured the comparison group: ‘I make friends with people from other races’. Given that this is one study, which has not been evaluated for medium to long term sustainability or impacts, it can only be described as ‘promising’ (Sherman et al., 1997).

Given the low number of studies identified, Mazerolle et al. (2020) also provided a summary of a further small sample of studies reporting on interventions that aligned with the review topic but did not meet the inclusion criteria due to weak evaluation designs. These studies illustrate a range of community policing‐based approaches being used by the police, such as recreation and sports activities, and community education and engagement around countering violent extremism and related topics. Individually, none of these studies were rigorous enough to be included in the review, but they do provide some indication that a range of community policing tactics may be useful as a part of an overall strategy to prevent terrorism and radicalisation to violence.

## 2 POLICE‐INVOLVED MULTIAGENCY PARTNERSHIPS THAT SEEK TO REDUCE RADICALISATION TO VIOLENCE

The second review by Mazerolle et al. (2021) focused on multiagency partnerships involving police to foster collaboration and reduce radicalisation to violence. This review is also clearly very relevant to community policing. Similar to the first study, this review also found a lack of clear evidence, but it also took an important step by adding a qualitative synthesis of research about how the intervention works (mechanisms), about intervention context (moderators), and about implementation factors and economic considerations.

As with Mazerolle et al. (2020), there was only one study assessed the impact of a police‐involved multiagency partnership on radicalisation to violence. This was the same Williams et al. (2016) study that we have set out above. Four studies met the inclusion criteria to assess the impact of a police multiagency partnership on interagency collaboration, as opposed to the outcome criteria of reducing radicalisation. Twenty‐six studies met the threshold for more thorough examination of the processes that facilitate or constrain implementation, as well as providing information about the costs and benefits of the programme.

Taken together the themes that emerged include the importance of taking time to build trust and shared goals among partners; not overburdening staff with administrative tasks; targeted and strong privacy provisions in place for intelligence sharing; and access to ongoing support and training for multiagency partners. In short, a set of leadership and implementation processes were identified as important considerations for the design and delivery of effective partnerships.

## 3 MENTAL HEALTH DIFFICULTIES AND RISK OF INVOLVEMENT IN TERRORISM

In considering the types of intervention and multi‐agency partnerships that might be effective as a part of a wider community policing approach, it is clearly important to understand the risk and protective factors in more detail. There has been an increasing focus on the potential role of mental health difficulties in the process of violent radicalisation into terrorism. In part, this has been fuelled by studies appearing to show high prevalence rates in some samples of terrorists. However, findings are inconsistent, with some studies reporting higher rates than those observed in the general population, some lower, and others that are comparable to those observed in the general population. Sarma et al. (2022) systematically reviewed this area, and they found no support for the mental health‐terrorism hypothesis. However, the review also suggested that there was some evidence of higher rates among some terrorist samples than others, notably among lone‐actor terrorists. This more nuanced and complex finding may indicate a key theme which comes through in a number of the reviews: that, whilst generalised findings can provide a useful starting point, it is important, as far as the range of studies allows, to explore sub‐groups within the data.

## 4 RISK FACTORS FOR COGNITIVE AND BEHAVIOURAL RADICALISATION

Wolfowicz et al. (2021) reviewed cognitive and behavioural risk and protective factors for radicalisation in democratic countries. Risk and protective factors, which increase or decrease the likelihood of these radicalisation outcomes, are used in risk assessment and counter‐radicalisation interventions. The selection of factors is often not evidence based. As a result, policies and practices are unlikely to be as effective as they could be, and can even increase stigmatisation of certain communities, thereby increasing the risk of radicalisation.

Wolfowicz et al. (2021) found that some of the factors most central to risk assessment and counter‐radicalisation interventions have relatively small relationships with radicalisation outcomes. Conversely, factors known to be associated with ordinary criminal outcomes have the largest relationships. These findings suggest the need for moving towards weighted risk assessment instruments, and alternative interventions. The findings of differences in the magnitude of the effects for different factors according to regional context suggest that risk assessment and interventions may be tailored to local contexts.

The findings of this systematic review that factors leading to non‐terrorist crimes may be significant predictors of radicalisation and violence seems to suggest that some policing strategies such as community policing, supported by appropriate risk assessments, may be capable of effective deployment to prevent these crimes too.

## 5 FAMILY FACTORS FOR RADICALISATION

Whilst Sarma et al. (2022) found no overall support for the mental health hypothesis, Zych and Nasaescu (2022) found that ‘parental ethnic socialization, having extremist family members and family conflict increase the risk of radicalization, whereas high family socio‐economic status, bigger family size, and high family commitment are protective factors’ (p.1).

There is reason to believe that families can be crucial to radicalisation to violence. Group influence on individual action is a well‐known phenomenon, and families are the most important social groups for many individuals. Transmission of antisocial behaviour from parents to children has been confirmed in several studies, mostly explained by the fact that children learn by observing and imitating their parents. Parenting styles are also known to have short and long‐term impact on children's lives. Thus, family‐related factors could be crucial to explain radicalisation to violence, but most of the empirical studies in the field include a limited number of participants and variables.

These reviews are significant for policy and practice because if risk and protective factors against violent radicalisation are discovered using rigorous scientific methods, interventions can be designed to focus on decreasing those risks and increasing protective factors. It is also crucial to identify the impact of violent radicalisation on families so that this could be mitigated.

Zych and Nasaescu found that parental bias and mistrust towards other cultures, having extremist family members and family conflicts were related to more radicalisation. High family socio‐economic status, bigger family size and family commitment were related to less radicalisation. The review also describes family‐related factors separately for cognitive and behavioural radicalisation, and for different radical ideologies such as Islamist, right‐wing and left‐wing. The results of this systematic review confirm the importance of families, although they should be interpreted with caution, taking into account a relatively low number of studies per analysis. Family factors are among the most important predictors of delinquency in general, and this also seems to be true for radicalisation to violence, but evidence is still limited and more studies on family‐related risk and protective factors are needed in this field.

## 6 COUNTER‐NARRATIVE INTERVENTIONS RELATED TO VIOLENT RADICALISATION

Carthy et al. (2020) reviewed the effectiveness of ‘counter‐narratives’, or targeted interventions that challenge the rationalisation(s) of violence, which may affect certain risk factors for violent radicalisation, including realistic perceptions of threat, in‐group favouritism, and out‐group hostility. The authors found little evidence that counter‐narrative interventions are effective at targeting primary outcomes related to violent radicalisation, such as behavioural intention to engage in manifestations of violent extremism, including terrorism. However, the scarcity of sufficient, high‐quality studies measuring these outcomes means that this evaluation cannot, yet, be regarded as conclusive and, indeed, may change with the emergence of further, rigorous research.

The authors did, however, find some evidence that counter‐narratives can be effective at targeting certain, secondary risk factors for violent radicalisation, including perceived group threat, in‐group favouritism, and out‐group hostility. However, across different intervention components, the effects are somewhat mixed, and may change with the emergence of new evidence.

Overall, the findings from the review do support the feasibility of the concept of using narrative‐based approaches for prevention, but highlight the care and complexity needed to design and implement effective counter‐narratives in the context of violent radicalisation. More specifically, using counter‐stereotypical exemplars, alternative narratives and inoculation techniques (eliciting resistance through the production of counter‐arguments) were all found to reduce overall risk factors for violent radicalisation. Persuasion did not have a significant effect. Such findings could be highly significant for the design and implementation of community policing strategies, because clarity, honesty of purpose and targeting of messaging could be an important strand to consider.

## 7 THE EVIDENCE AND GAP MAP (EGM)

The commissioning of an EGM was a key part of the Campbell/5RD CVEN programme, designed both to map existing research knowledge and to provide a guide for future review areas and priorities for primary research.

The search for the EGM (Sydes et al., 2023) was extensive: nearly 70,000 unique records, which, after screening were reduced to 67 studies eligible for the EGM (from 58 documents). These included 2 systematic reviews, 14 randomised controlled trials and 51 quasi‐experimental studies. One limitation was that the majority of studies were conducted in the United States and Global North. There were no eligible impact evaluations carried out in Central or South America, Oceania, Sub‐Saharan Africa or Northeast Asia.

The strongest evidence was for policing interventions (more than 50 studies) or multi‐agency partnerships that included the police as a partner. However, as we have seen above, even in some of these areas, the evidence remains reliant on too small a number of studies. However, evidence for courts or custodial corrections interventions was even more limited and there were no eligible studies reported on community corrections interventions for preventing terrorism/radicalisation.

The EGM highlighted that a wide variety of outcome measures had been used in the evaluations – an issue that had already been flagged by the 5RD CVEN. The most commonly assessed outcomes were measures of terrorism, investigation efficacy and organisational factors. There was very limited research which assessed intervention effectiveness against measures of violent extremism and/or radicalisation to violence.

## 8 CONCLUDING THOUGHTS AND FUTURE PROSPECTS

With a further eight reviews due to be completed and published in the first 4‐year programme, the Campbell 5RD programme is building one of the most important bodies of systematically reviewed evidence on CVE, and making it available for the world. This was the ambition from the outset, but as the programme started, there were concerns that there might be too many empty reviews, like Lum et al.'s (2006) original review of counter‐terrorism. As the programme has developed and stretched the boundaries of the systematic review process, it is encouraging, as the brief overview above has shown, that the programme is delivering the evidence that it was designed to deliver, as well as highlighting areas for more focus for primary research and evaluation.

## CONFLICT OF INTEREST STATEMENT

The authors declare no conflicts of interest.
